# Post-traumatic Stress Disorder With Flashbacks of an Old Childhood Memory Triggered by Right Temporal Lobe Epilepsy Surgery in Adulthood

**DOI:** 10.3389/fpsyt.2020.00351

**Published:** 2020-04-28

**Authors:** Antoine Yrondi, Luc Valton, Viviane Bouilleret, Nozar Aghakhani, Jonathan Curot, Philippe Jean Birmes

**Affiliations:** ^1^Service de Psychiatrie et de Psychologie Médicale, Centre Expert Dépression Résistante FondaMental, CHU de Toulouse, Hôpital Purpan, ToNIC, Toulouse NeuroImaging Center, Université de Toulouse, Inserm, UPS, Toulouse, France; ^2^Explorations Neurophysiologiques, Hôpital Pierre Paul Riquet, CHU Purpan, Toulouse, France; ^3^Centre de Recherche Cerveau et Cognition, University of Toulouse, Centre National de la Recherche Scientifique CerCo, Toulouse, France; ^4^Service de Neurophysiologie Clinique et D'épileptologie, Hôpitaux Universitaires Paris-Sud, Assistance Publique-Hôpitaux de Paris, Le Kremlin-Bicêtre, France; ^5^Service de Neurochirurgie, Hôpitaux Universitaires Paris-Sud, Assistance Publique-Hôpitaux de Paris, Le Kremlin-Bicêtre, France; ^6^Toulouse NeuroImaging Center, Université de Toulouse, Inserm, UPS, Toulouse, France

**Keywords:** PTSD (post traumatic stress disorder), epilepsy, surgery, hospital, childhood trauma, CTQ

## Abstract

**Background:**

A plethora of data show that the hippocampus and the amygdala are involved in post-traumatic stress disorder (PTSD). Neural dysfunctions leading to PTSD (e.g. how the amygdala and the hippocampus are altered) are only partially known. The unusual case of a patient presenting with refractory epilepsy and developing PTSD immediately after surgery is described. Such symptoms in epileptic patients may help to explore PTSD mechanisms.

**Case report:**

A 41-year-old male suffering from partial refractory temporal lobe epilepsy was operated in May 2017. A right amygdala, hippocampus, and temporal pole selective resection was performed. He experienced intense PTSD symptoms 1 month after surgery. He complained about repetitive intrusive memories of abuse. The PTSD checklist score was equal to 62/80. He reported a history of childhood abuse: physical and emotional abuse as well as emotional negligence, assessed with the Childhood Trauma Questionnaire. No other medical history was recorded. He never complained about PTSD or any other psychiatric symptoms before surgery.

**Conclusion:**

this case indicates that PTSD may occur after temporal lobe epilepsy surgery and may specifically stem, as in this context, from the excision of part of the medial temporal lobe structures. Although rarely reported, PTSD may be undiagnosed when not selectively detected *via* multi-disciplinary neurological and psychiatric management, in the preoperative period and the immediate and delayed postoperative period.

## Background

Post-traumatic stress disorder (PTSD) is defined (1) as exposure to a traumatic event that is persistently re-experienced. Subjects avoid trauma-related stimuli and suffer from negative thoughts or feelings from trauma-related hyperarousal and reactivity. Symptoms last at least 1 month and create distress or functional impairment. A wealth of data shows that the hippocampus and the amygdala are involved in PTSD (1). The hippocampus plays a key role in encoding and recalling episodic memories as well as in triggering contextual, spatial, and temporal aspects of memories (2). The amygdala plays a cardinal role in threat detection, fear response, and emotional memory (3). It is crucial in regulating the memory of stressful events, particularly in fear-conditioning (4). Over-activation of the amygdala appears to participate in defective mechanisms of fear extinction and over-consolidation of fear-conditioning, resulting in the persistence of fear responses in PTSD (5). It may also underlie long-term intrusion symptoms and hyperreactivity (6).

However, neural dysfunctions leading to PTSD (e.g., how the amygdala and hippocampus are altered) are as yet not entirely known. In this context, symptoms in epileptic patients are invaluable for exploring PTSD mechanisms. Exposure to psychological trauma is frequent in epilepsy (7).Moreover, the risk of PTSD in patients presenting with epilepsy is higher than in those without it (odds ratio: 2.0, 95% CI: 1.2–3.3) (8). Epilepsy surgery is an effective treatment for patients presenting with refractory epilepsy (9). Nevertheless, psychiatric complications are reported in 30% to 40% of patients following this surgery (10).

The unusual case of a 41-year-old male presenting with refractory epilepsy and developing PTSD immediately after surgery is outlined. The patient experienced pathological flashbacks of an old trauma unrelated to the surgical experience or epilepsy. Surgical resection limited to the medial temporal lobe (MTL) and temporal pole appeared to be the only factor explaining the onset of PTSD. This case—that to our knowledge is unlike any other in literature—raises questions about the role of MTL structures in the induction of PTSD and in the inhibition of traumatic events. It also raises concerns about the impact of the partial removal of the limbic system on the physiopathology and the delayed onset of PTSD symptoms. Moreover, this observation highlights the need for a thorough psychiatric investigation prior to surgery, that is not only focused on mood or psychotic disorders. The use of clinical data was approved by Toulouse University Hospital Ethics Committee (MR003). Informed consent was obtained from the patient for the publication of this case report.

## Case Report

The patient had been suffering from partial refractory TLE since the age of 9 years. He had no history of febrile convulsions, other brain injuries or psychiatric disorders. Partial seizures were associated with loss of contact and exacerbated epigastric pain, usually accompanied by rotation of the head and the eyes to the left and postictal amnaesia. Seizures had been transiently controlled with oxcarbazepine between 2011 and 2016. Regrettably, they reappeared and became more frequent in 2016, despite modified and increased medical treatment.

**Figure 1:**
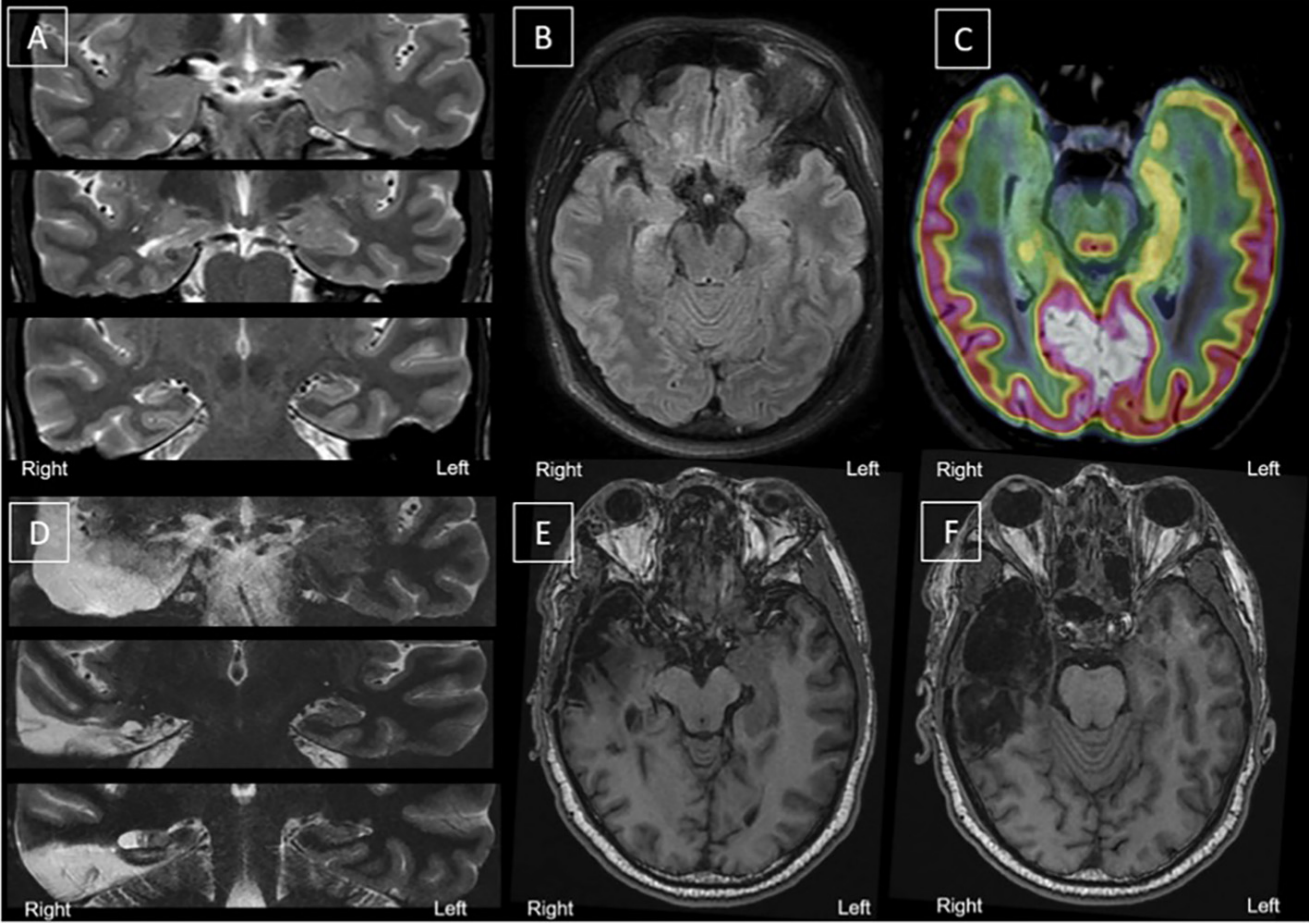
Morphological and metabolic imaging before and after surgery. **(A–C)** Before surgery. **(D–F** After surgery. **(A)**: Coronal slices, top to bottom, anterior to posterior hippocampus—T2 MRI; **(B)** Axial Slices (T2 MRI) showing right hippocampal sclerosis; **(C)**: Fusion between T2 MRI (hippocampal axis) and FDG-PET (hippocampal axis) showing right medial hypometabolism. **(D)** Coronal slices, top to bottom, anterior to posterior hippocampus—T2 MRI; **(E–F)** Axial slices (T1 MRI) of the right medial temporal area after selective surgery. Surgery left a porencephalic cavity with gliosis, limited to the right temporal region; right meningeal thickening and T1 contrast enhancement near amygdalo-hippocampal area (not shown). FDG-PET, F-18-fluorodesoxyglucose positron emission tomography; MRI, magnetic resonance imaging.

A pre-surgical evaluation was performed. A brain magnetic resonance imaging (MRI) scan highlighted a right hippocampal sclerosis (**Figures 1A, B**). No other lesions were observed. Long-term scalp video-electroencephalography (EEG) monitoring recorded two typical partial seizures with right temporal ictal discharge, interictal epileptiform discharges (IED), chiefly located in the right temporal region, and few left temporal asynchronous IEDs. The F-18-fluorodesoxyglucose positron emission tomography (FDG-PET) scan showed hypometabolism limited to the mesial and polar parts of the right temporal lobe (**Figure 1C**). The patient was operated in May 2017. A right amygdala, hippocampus, and temporal pole selective resection was performed (**Figures 1D–F**). The pathological examination confirmed the diagnosis of hippocampal sclerosis.

No complications were observed during the procedure or in the immediate post-operative period. During the immediate post-operative follow-up, the patient reported a feeling of malaise and anxiety, interpreted initially as mixed anxiety-depressive syndrome. Psychotherapy was proposed to the patient. Due to a persistent state of malaise, PTSD was finally diagnosed by a psychiatrist. The patient experienced intense PTSD symptoms one month after surgery. He complained of repetitive intrusive memories of abuse. He described seeing a family member entering his bedroom to abuse him, generating repetitive, involuntary, and invasive memories of sustained abuse. He felt as if these episodes of abuse were starting again, thus, exacerbating his feelings of distress.

He reported a history of abuse between the ages of 9 and 23 years. The Childhood Trauma Questionnaire (11) revealed emotional abuse (score = 22), severe physical abuse (score = 18) and severe emotional negligence (score = 19). No sexual abuse was reported, and no other history was recorded. Interestingly, he had never complained about PTSD symptoms linked to these events before surgery.

Various environmental clues triggered the sudden involuntary retrieval of memories. “When I hear my wife's keys in the door, I have flashbacks from when I used to be abused with keys by somebody entering the house.” He reported nightmares associated with these events and avoided situations that could make those memories resurface. Neurovegetative signs of fear (tachycardia, excessive perspiration) and hypervigilance accompanied these reviviscences. He described a persistent negative, emotional state with feelings of anger and fear, resulting in a diminished interest in his daily activities. He also experienced difficulties in falling asleep, irritability, attention disorder, and poor concentration. The PTSD checklist score (PCL-5)(12) was equal to 62/80 (score ≥ 32: “probable” PTSD).

A post-operative scalp-EEG did not display any IEDs or focal slow-down. Six months after surgery, the follow-up MRI showed only temporal post-operative sequelae (**Figures 1D–F**) and very limited residues of the amygdala and the remaining posterior part of the hippocampus (**Figure 1**). The patient was still entirely seizure-free (ILAE class 1) and EEG was normal 19 months after surgery (last follow-up).

Unfortunately, eight months after the procedure, intrusive memories persisted with a feeling that abuse was recommencing. The patient still relied on strategies to avoid situations that would remind him of traumatic events. He reported persistent sleeping disorders with hypervigilance, outbursts, irritability, and little interest in his usual activities. PCL5 score was equal to 42/80. There was evidence of a negative social impact with signs of withdrawal and troubles in his conjugal relationship. Treatment with SSRI medication (sertraline 125 mg/day) improved clinical symptoms after 2 (PCL5: 23) and 4 (PCL5: 16) months of follow-up. Because of persistent PTSD symptoms and a partial response to medication, cognitive behavioral therapy (CBT) was introduced. Trauma-focused CBT was employed, which included exposure and cognitive techniques (such as cognitive restructuring), exposure to the traumatic memory by using imaginal exposure, writing down the traumatic narrative, and reading the traumatic memory out loud (13–15). Cognitive restructuring focused on teaching the patient to identify dysfunctional thoughts and thinking errors, to elicit rational alternative thoughts and to reappraise beliefs about himself, the trauma and the world (13–15).

## Discussion

To our knowledge, this is only the second case of reported PTSD following epilepsy surgery (16) and the first case detailing isolated PTSD in the absence of dissociative motor disorders, such as psychogenic non-epileptic seizures. A few studies report a decrease in left amygdala volume associated with abuse-related PTSD and a smaller total hippocampal volume in abused subjects presenting with high PTSD symptom severity as compared to those presenting with low-PTSD severity level (17–22). In our patient, the pre- and post-operative MRI did not show obvious left amygdala and left hippocampus volume reduction, whereas the right hippocampus was atrophic prior to resection.

Based on this report, two major assumptions can be postulated about PTSD development, which are not mutually exclusive and may coexist: 1) a certain resilience (ability to recover from stress, adversity, failure, challenges or even trauma) before surgery supported by a balance between both hemispheres; 2) a dysregulation of emotional and memory neural networks after the removal of some of their key components and a release following temporal resection.

Considering the first hypothesis, Adami et al. (16) assume the possibility of PTSD existing prior to surgery, albeit masked by concomitant disorders. Although our case is a retrospective report, there is no sign of either total or partial PTSD or adaptation disorders prior to epilepsy surgery. Furthermore, there seem to be no signs of any other psychiatric comorbidity. These observations do not favor such a hypothesis. However, this does not exclude hypothetical good resilience and the repression of PSTD symptoms prior to surgery or the fact that PTSD symptoms may not occur until years after the event (23). A comparison of resting-state fMRI connectivity before and after surgery in TLE patients highlighted a pre-surgical resilience network, which is more developed in patients displaying no post-surgical episodes (as in the case of our patient) (24). Therefore, it can be assumed that epilepsy surgery engendered the reorganisation of functional brain networks, rendering this resilience network dysfunctional.

Another hypothesis could be the dysregulation of emotional and/or memory neural networks after the removal of some of their key components. TLE is considered to be a functional connectivity disorder (25). Several studies confirm functional changes in the temporal lobe following epilepsy surgery (24, 25). In our patient, selective MTL resection and the loss of some major hubs of memory and emotion circuits may have resulted in their abnormal activation (26), with an excess of involuntary memory retrieval and emotional valence. There is a possibility that the residual amygdala and the posterior hippocampus which were not removed through surgery are only disconnected, thus still functional. Indeed, the remaining amygdala (heterogeneous structure with numerous nuclei, each with various connections) and the posterior hippocampus were disconnected from each other. Nevertheless, each of these two structures were still connected with core nodes of episodic memory networks (for instance, the hippocampus with the posterior cingulate cortex, the amygdala with the orbitofrontal cortex) with a probable residual influence on the global networks. Pre-operative FDG-PET imaging suggests a non-functional right [portion of the] amygdala and a very limited preserved metabolism of the posterior part of the right hippocampus. However, a lasting functioning of these residual structures would still reflect the hypothesis of connectivity remapping within memory neural networks after surgery.

It could be argued that surgery alone may be a major stressor. Although careful conclusions must be drawn with respect to a unique case, neuroimaging evidence, both in literature and in this case, report fit the general hypothesis of a release of inhibition favoring the revelation of latent PTSD following MTL resection. This hypothesis could account for the delayed onset of PTSD, stemming from repeated childhood trauma, secondary to TLE surgery. A more developed resilience network associated with abrupt changes in functional connectivity in memory and emotion networks may be the main factors behind the occurrence of PTSD.

Lastly, this case refers to the old debate on the permanence of our memories across time and raises the crucial question: how does one manage memories resurfacing after years? Are these true memories successfully retrieved in therapy? Are these unwittingly planted false memories or reconstructions biased/distorted by unconscious influences (27–29)? Are they symbolic expressions, historically false but representing some deep underlying truth (27)? Nevertheless, in addition to the memories, in this case, PTSD symptoms occurred after some years. Moreover, a significant number of people witnessed this child abuse, thus confirming the content and truthfulness of the reported memories.

## Conclusion

This case emphasizes that PTSD may occur after TLE surgery and may specifically be caused, within this context, by the excision of a part of the MTL structures. Although rarely reported, PTSD may be misdiagnosed when not selectively detected *via* multi-disciplinary neurological and psychiatric management. The possibility of a traumatic life event and PTSD symptoms should be routinely considered following epilepsy surgery, as well as psychotic and mood symptoms.

## Data Availability Statement

The data sets generated for this study are available on request to the corresponding author.

## Ethics Statement

The studies involving human participants were reviewed and approved by Toulouse University Hospital Ethics Committee (MR003). The patients/participants provided their written informed consent to participate in this study.

## Author Contributions

All authors equally took part in the drawing up of this paper. AY and PB conducted the psychiatric assessment. LV and JC carried out the post-operative neurological assessment. VB carried out the pre-operative neurological assessment. NA performed the surgery.

## Conflict of Interest

AY has received speaker honoraria unrelated to the present work from AstraZeneca, Janssen, Lundbeck, Otsuka, Servier.

The remaining authors declare that the research was conducted in the absence of any commercial or financial relationships that could be construed as a potential conflict of interest.

## Abbreviations

CBT, Cognitive Behavior Therapy; FDG-PET, F-18-fluorodesoxyglucose Positron Emission Tomography; IED, Interictal EpileptiformDischarge; PCL, Post-traumatic Checklist; PTSD, Post-traumatic Stress Disorder; TLE, Temporal Lobe Epilepsy; MTL, Medial Temporal Lobe.
